# On partial likelihood and the construction of factorisable transformations

**DOI:** 10.1007/s41884-022-00068-8

**Published:** 2022-06-28

**Authors:** H. S. Battey, D. R. Cox, Su Hyeong Lee

**Affiliations:** 1 Department of Mathematics, Imperial College London, London, UK; 2 Nuffield College, University of Oxford, Oxford, UK; 3 Mathematical Institute, University of Oxford, Oxford, UK

**Keywords:** Inferential separation, Marginal likelihood, Matched comparisons, Method of characteristics, Partial differential equations, Nuisance parameters

## Abstract

Models whose associated likelihood functions fruitfully factorise are an important minority allowing elimination of nuisance parameters via partial likelihood, an operation that is valuable in both Bayesian and frequentist inferences, particularly when the number of nuisance parameters is not small. After some general discussion of partial likelihood, we focus on marginal likelihood factorisations, which are particularly difficult to ascertain from elementary calculations. We suggest a systematic approach for deducing transformations of the data, if they exist, whose marginal likelihood functions are free of the nuisance parameters. This is based on the solution to an integro-differential equation constructed from aspects of the Laplace transform of the probability density function, for which candidate solutions solve a simpler first-order linear homogeneous differential equation. The approach is generalised to the situation in which such factorisable structure is not exactly present. Examples are used in illustration. Although motivated by inferential problems in statistics, the proposed construction is of independent interest and may find application elsewhere.

## Introduction

Parametric statistical inference in models with many parameters relative to the number of observations raises issues that are at least implicitly differential geometric. For inference on a scalar or vector interest parameter $\psi$, the profile log-likelihood function for $\psi$ replaces nuisance parameters by their constrained maximum likelihood estimates. The approach may give highly miscalibrated inference for $\psi$ when the number of nuisance parameters is large relative to the amount of information in the sample, and considerably suboptimal inference for even moderately many such parameters. Three broad approaches are to apply an interest-respecting orthogonal reparameterisation, to base inference on an adjusted version of the signed-likelihood root called $r^*$, or to construct a suitable partial likelihood.

Parameter orthogonalisation, proposed by [10] and based on the solution to a set of partial differential equations, leads to higher-order accuracy of nuisance maximum likelihood estimators in a moderate deviation range of $\psi$, and thereby removes the leading-order bias term in the maximum likelihood estimator of $\psi$. This can be observed from the expansions on p.150 of [1] on noting that parameter orthogonalisation, by definition, sets relevant blocks of the Fisher information matrix to zero. Two asymptotically equivalent versions of $r^*$ are due to [2, 21], the latter using implicit conditioning on an approximately ancillary statistic. Partial likelihood, formalised to some extent by Cox [9], uses only part of the full likelihood function for inference on $\psi$, possibly relinquishing some information. All three approaches, when available, often yield remarkably accurate inference when the amount of information is small, or nuisance parameters are numerous.

Cox [9] stated five problems associated with partial likelihood, of which the first was: “to provide constructive procedures for finding useful partial likelihoods”. The problem remains open, and the purpose of the present paper is to provide a modest step towards addressing it, focussing primarily on a class of matched comparison problems. Section 2 gives a formalisation of inferential separation based on partial likelihood, while Sect. 4 presents a version suitable for matched comparison settings.

## Partial likelihood and inferential separation

Suppose the outcomes are realisations of random variables $Y_1,\ldots ,Y_b$ whose joint distribution depends on parameters $(\psi ,\lambda )$. The notation *b* in place of the more conventional *n* is for consistency with the rest of the paper, in which *n* is the total sample size, $Y_i$ is a vector of size *m*, and $b=n/m$ represents the number of blocks of size *m*. In the present section such block structure is not needed.

Consider inference on an interest parameter $\psi$ from a parameter-based factorisation of the full likelihood function $L(\psi ,\lambda ;y)$ of the form

2.1 $$\begin{aligned} L(\psi ,\lambda ;y)=L_{\text {pa}}(\psi ;y)L_{\text {r}}(\psi , \lambda ;y). \end{aligned}$$

The factor $L_{\text {pa}}(\psi ;y)$ is called the partial likelihood Cox [9]. Ideally, little or no information for inference on $\psi$ is lost through relinquishment of the remainder likelihood $L_{\text {r}}(\psi , \lambda ;y)$.

A factorisation of the form (2.1) can be induced in various ways, as is apparent from the examples in Cox [9]. The important distinction between factorisations on the parameter space and those on the sample space is discussed in detail by [11, 12]. The present paper is concerned with factorisations on the parameter space induced through sample-space factorisations of the marginal and conditional types.

Let (*S*, *R*, *A*), where *A* is ancillary, be a jointly sufficient statistic for $(\psi ,\lambda )$ based on $Y=(Y_1,\ldots ,Y_b)$, where any of $Y_i$, $\psi$ or $\lambda$ may be vectors. An ancillary statistic need not be available. When it is, (*S*, *R*, *A*) is treated as minimal sufficient.

The full likelihood function for $(\psi ,\lambda )$ is the density function $f_Y$ of *Y*, viewed as a function of the parameters with *y* fixed at the observed value of *Y*:

$$\begin{aligned} f_{Y}(y;\psi ,\lambda )\propto f_{S,R,A}(s,r,a;\psi ,\lambda ) = f_A(a) f_{S \mid A}(s \, |\, a; \psi , \lambda )f_{R\mid S, A}(r \, |\, s, a; \psi , \lambda ). \end{aligned}$$

That it may be beneficial, or indeed necessary, to use only part of the likelihood function for inference on $\psi$ was noted by [3], who proposed conditional likelihood based on $f_{R\mid S, A}$ when this quantity is free of $\lambda$. Another special case is $f_{S \mid A}(s \, |\, a; \psi , \lambda )=f_{S | A}(s \, |\, a; \psi )$ so that one choice of $L_{\text {pa}}(\psi ;y)$ is $f_{S | A}(s \, |\, a; \psi )$. Similarly, if

$$\begin{aligned} f_{S \mid A}(s \, |\, a; \psi , \lambda )=g_1(s,a;\psi )g_2(s,a;\psi ,\lambda ), \end{aligned}$$

a $\lambda$-free partial likelihood for $\psi$ can be constructed as $L_{\text {pa}}(\psi ;y)=g_1(s,a;\psi )$. In the absence of an ancillary statistic, $f_{S \mid A}(s \, |\, a; \psi , \lambda )=f_{S}(s; \psi , \lambda )$, and when this is free of $\lambda$, $L_{\text {pa}}(\psi ;y)=f_{S}(s; \psi )$ is called the marginal likelihood function [18, 22].

## Matched comparison problems

Matched comparison studies in blocks of size *m* represent a popular and effective method of experimentation for the assessment of *m* treatment effects, of which one typically represents a control or base treatment. Such designs entail matching *b* sets of *m* individuals based on their intrinsic features before randomising each treatment to one unit per block.

With $m=2$ it is convenient to write $(Y_i)_{i=1}^b=(T_i, C_i)_{i=1}^b$ for outcomes on the treated and untreated individuals in each of the *b* pairs. The distributions of $T_i$ and $C_i$ are typically assumed equal up to the presence of a treatment parameter, $\psi$, and known modulo a pair-specific nuisance parameter, $\lambda _i$. Inclusion of a nuisance parameter per pair avoids having to specify in detail those aspects of the probabilistic model that are not of primary subject-matter importance. The nuisance parameters may, for example, encompass the effects of unmeasured covariates in the form $\lambda _i=h(x_i^{\mathrm{T}}\beta )$. It also makes the assumption of independence of $T_i$ and $C_i$ reasonable. There are limitations to this approach. Notably non-constancy of the treatment effect would not be directly detectable through consideration of all pairs simultaneously.

A key consideration is whether the nuisance parameters $\lambda _1,\ldots ,\lambda _b$ are to be treated as realisations of random variables or as fixed arbitrary constants. Appendix A formalises the two formulations and argues in favour of the latter. While conceptually compelling, the analysis for fixed nuisance parameters is more challenging and context-dependent. When available, it tends to be based on a form of partial likelihood obtained from distributional factorisations on the sample space: either marginal or conditional likelihood. Motivated by this, the present paper seeks a function $s(Y_i)$ of the components of $Y_i$ such that the density function of $S_i=s(Y_i)$ depends on $\psi$ but not on $\lambda _i$. We call $S_i$ a factorisable transformation of $Y_i$. A function *s* inducing such factorisable structure need not exist, raising the question of whether useful approximate versions may be found.

For larger values of *m*, typically $\psi =(\psi _1,\ldots ,\psi _m)^{\mathrm{T}}$ represents a vector of treatment effects, and we let $Y_{i1},\ldots ,Y_{im}$ represent the independent outcome variables associated with each treatment in the *i*th block, $i=1,\ldots ,b$.

The formulation of matched comparison problems as inducing large numbers of nuisance parameters for inference on a scalar treatment effect was presented by [7], and has been discussed from various perspectives by [1, 4, 13, 23–25, 27]. The following results illustrate some of the difficulties.

### Example 1

Bartlett [3] noted that when two random variables, $Y_{i1}$ and $Y_{i2}$ say, are normally distributed of mean $\mu _i$ and variance $\sigma ^2$ for $i=1,\ldots , b$, the maximum likelihood estimator of $\sigma ^2$ converges in probability to $\sigma ^2/2$.

### Example 2

Let $(T_i,C_i)_{i=1}^b$ be independent pairs of independent binary variables, each valued in $\{0,1\}$, and let

$$\begin{aligned} \text {pr}(C_i=1) = \frac{e^{\lambda _i}}{1+e^{\lambda _i}}, \quad \text {pr}(T_i=1) = \frac{e^{\lambda _i+\psi }}{1+e^{\lambda _i+\psi }}. \end{aligned}$$

The interest parameter $\psi$ is the logistic difference between the probabilities. Cox [7] indicated the appropriate conditional analysis, while [1], example 4.6 demonstrated difficulties with direct use of the likelihood function for estimation of $\psi$, showing that the estimator converges in probability to $2\psi$.

### Example 3

Suppose that $T_i$ and $C_i$ are independently exponentially distributed of rates $\lambda _i \psi$ and $\lambda _i/\psi$ respectively for $i=1,\ldots ,b$. Thus $T_i$ and $C_i$ have a constant hazard ratio $\psi ^2$. A slightly different parameterisation of this example was used by [24, 25].

The likelihood equations for $\hat{\psi }$ and $\hat{\lambda }_1,\ldots ,\hat{\lambda }_b$ are

$$\begin{aligned} 0= & {} \nabla _{\lambda _i} \ell (\hat{\psi },\hat{\lambda }_1,\ldots ,\hat{\lambda }_b) = 2\hat{\lambda }_{i}^{-1}-(T_i \hat{\psi } + C_i/\hat{\psi }),\\ 0= & {} \nabla _{\psi } \ell (\hat{\psi },\hat{\lambda }_1,\ldots ,\hat{\lambda }_b) = - \sum _{i=1}^b \hat{\lambda }_i T_i + \sum _{i=1}^b\hat{\lambda }_i C_i/\hat{\psi }^2. \end{aligned}$$

Thus, on substituting $\hat{\lambda }_i=2(T_i \hat{\psi } + C_i/\hat{\psi })^{-1}$ into the likelihood equation for $\hat{\psi }$ and simplifying, the maximum likelihood estimator $\hat{\psi }$ is the solution in $\psi$ to

3.1 $$\begin{aligned} 0= \sum _{i=1}^b \frac{C_i/\psi - T_i\psi }{C_i/\psi + T_i\psi }. \end{aligned}$$

Lindsay [25] established that this estimator of the constant hazard ratio is consistent and asymptotically normally distributed as $n=2b \rightarrow \infty$. This is in contradistinction to the seemingly similar Examples 1 and 2. However, the usual estimator of variance of $\hat{\psi }$ is miscalibrated. Lindsay [25] recommended a treatment in which $\lambda _1,\ldots ,\lambda _b$ are regarded as independent and identically distributed random variables from a parametric distribution of known form. Mispecification of the form can lead to major difficulties.

We favour a transformation to $S_i=T_i/C_i$, whose density function at $s>0$ is $\psi ^{2}/(1+\psi ^{2}s)^2$ producing the marginal likelihood,

$$\begin{aligned} L_{\text {pa}}(\psi ;y)=\prod _{i=1}^b f_{S}(s_i;\psi ), \end{aligned}$$

as a fruitful choice of partial likelihood. This is to be viewed as a function of $\psi$. Since $(S_i)_{i=1}^b$ are independent and identically distributed, consistency of the marginal likelihood estimator $\hat{\psi }_{\text {m}}$ is expected in view of standard maximum likelihood theory, although verification of the usual regularity conditions is complicated by non-existence of moments of $S_i$. The proof of Proposition 3.1 establishes consistency directly.

### Proposition 3.1

Let $\hat{\psi }_{\text {m }}$ be the marginal maximum likelihood estimator based on the transformed random variables $(S_i)_{i=1}^b$. Then $\hat{\psi }_{\text {m }}$ is consistent for $\psi$ as $b\rightarrow \infty$.

### Proof

The marginal likelihood equation for $\hat{\psi }_{\text {m}}$ is

3.2 $$\begin{aligned} 1 = \frac{2}{b}\sum _{i=1}^b \frac{\hat{\psi }^{2}_{\text {m}} S_{i}}{1+\hat{\psi }^{2}_{\text {m}} S_{i}}. \end{aligned}$$

A strong law of large numbers implies that, for any $\kappa >0$,

$$\begin{aligned} \frac{1}{b}\sum _{i=1}^{b} \frac{\kappa ^{2}S_{i}}{1+\kappa ^{2}S_{i}} \rightarrow _{a.s.} \frac{\kappa ^2\{(\kappa -\psi )(\kappa +\psi )+2\psi ^{2}(\log \psi - \log \kappa )\}}{(\kappa ^2-\psi ^{2})^2}. \end{aligned}$$

Thus in the limit as $b\rightarrow \infty$, $\hat{\psi }_{\text {m}}$ satisfies

3.3 $$\begin{aligned} 1=\frac{2\hat{\psi }^{2}_{\text {m}}\{(\hat{\psi }_{\text {m}}-\psi )(\hat{\psi }_{\text {m}}+\psi )+2\psi ^{2}(\log \psi - \log \hat{\psi }_{\text {m}})\}}{(\hat{\psi }^{2}_{\text {m}}-\psi ^{2})^2}. \end{aligned}$$

The right hand side of (3.3) is 1 only in the limit as $\hat{\psi }_{\text {m}}\rightarrow \psi$.

It follows directly from the consistency established in Proposition 3.1 that $(\hat{\psi }_\text {m}-\psi )\{-\ell ^{\prime \prime }_{\text {pa}}(\hat{\psi })\}^{1/2}$ is asymptotically standard normally distributed, and similarly for the likelihood ratio statistic based on the marginal likelihood.

Conditional likelihood is available and fruitful in the matched comparison context if $(T_i,C_i)$ can be transformed bijectively to new variables, $(S_i,R_i)$ say, such that the conditional density function of each $S_i$ given $R_i=r_i$ depends on $\psi$ but not on $\lambda _i$. When available, this situation is typically easy to detect from inspection of the log-likelihood function, as it only requires identifying $R_i=r(T_i,C_i)$ as a sufficient statistic for $\lambda _i$. The statistic $S_i$ can then be chosen based on convenience of calculating the conditional density, provided that the transformation $(T_i, C_i)\rightarrow (S_i, R_i)$ is bijective.

Deducing a function $S_i=s(T_i,C_i)$ that gives a suitable marginal likelihood as in Example 3 is considerably more difficult and, to our knowledge, a systematic construction has not been attempted.

## A systematic construction of marginal likelihood

For ease of exposition the case of $m=2$ is discussed first, suppressing the pair index *i* on $\lambda _i$ and $(T_{i},C_i)$ except when it is necessary to be explicit.

To establish a transformation $(T,C)\rightarrow (S,R)$, write the transformation equations as $s=s(t,c)$, and $r=r(t,c)$. Since the transformation is assumed bijective, the inverse equations are $t=t(s,r)$ and $c=c(s,r)$. A transformation satisfying $f_{S}(s;\psi , \lambda ) = f_{S}(s;\psi )$ is sought, using only the joint probability density or mass function of *T* and *C*, denoted by $f_{T,C}(t,c;\psi ,\lambda )$. The probability function of an arbitrary transformed random variable $S=s(T,C)$ is expressible in terms of this either by specifying the Jacobian matrix of the transformation, or by using Laplace transforms. The latter is found to be more convenient. Thus consider

4.1 $$\begin{aligned} f_{S}(s;\psi , \lambda ) = \frac{1}{2\pi i} \int _{\tau - i \infty }^{\tau + i\infty } \exp \{z s(t,c)\} T_{\lambda }(s,z)dz, \end{aligned}$$

where ${s:\mathbb {R}^2 \rightarrow \mathbb {R}}$, $\tau$ is anywhere in the interval of convergence of the moment generating function of *S* and $T_{\lambda }(s,z)$ is the Laplace transform,

$$\begin{aligned} T_{\lambda }(s,z)=\int _{-\infty }^\infty \int _{-\infty }^\infty \exp \{-z s(x,y)\}f_{T,C}(x,y; \psi , \lambda ) dx dy, \quad z\in \mathbb {C}. \end{aligned}$$

Since (4.1) may only depend on $\lambda$ through $T_{\lambda }$, Battey and Cox [4] suggested choosing the function *s*(*t*, *c*) to make $T_{\lambda }$ independent of $\lambda$, identically in *z*, $\psi$ and $\lambda$. It is in fact sufficient by Cauchy’s theorem (e.g. [31, Sect. 5]) that independence be achieved only at points *z* of singularity, but this is more difficult and inconsequential unless the analytic continuation of the moment generating function of *S* has a singularity at zero, as will become clear from the discussion below.

A function *s* delivering independence of $\lambda$ is sought by differentiating $T_\lambda$ partially with respect to $\lambda$ and solving the resulting integral equation for *s*(*t*, *c*), identically in *z*, $\psi$, and $\lambda$. This equation is

4.2 $$\begin{aligned} \frac{\partial }{\partial \lambda }\int _{-\infty }^\infty \int _{-\infty }^\infty \exp \{-z s(t,c)\} f_{T,C}(t,c; \psi , \lambda ) dt dc= 0. \end{aligned}$$

Depending on the support of the distributions of *T* and *C*, the range of integration may be restricted.

The partial differential operator can be interchanged with the integral sign by Leibniz’s theorem but it is more fruitful to first apply a change of variables from (*t*, *c*) to (*w*, *v*), say, chosen such that all dependence on $\lambda$ is transferred from $f_{T,C}(t,c;\psi ,\lambda )$ to $s(w,v; \psi , \lambda )$. The condition required for this is that $f_{T,C}(t,c;\psi ,\lambda )$ is expressible as

4.3 $$\begin{aligned} f_{T,C}(t,c;\psi , \lambda )=\kappa \frac{d w(t)}{d t}\frac{d v(c)}{d c} g\{w(t,\psi ,\lambda ),v(c,\psi ,\lambda ),\psi \}, \end{aligned}$$

where $\kappa$ is a constant and *g* depends on $\lambda$ only through the bijective functions $w(t;\psi ,\lambda )$ and $v(c;\psi ,\lambda )$. Assuming this is satisfied, (4.2) is

4.4 $$\begin{aligned} \frac{\partial }{\partial \lambda }\int _{\mathcal {V}}\int _{\mathcal {W}} \exp [-z s\{t(w,\psi ,\lambda ),c(v,\psi ,\lambda )\}] g(w,v,\psi ) dw dv= 0. \end{aligned}$$

By Leibniz’s theorem, the chain rule and positivity of *g*, a sufficient and necessary condition for *s* to solve the nonlinear double integral equation (4.4) for any $z\ne 0$ is that it solves the first-order linear homogeneous differential equation:

4.5 $$\begin{aligned} \frac{\partial }{\partial \lambda } s\{t(w;\psi , \lambda ),c(v;\psi , \lambda )\} = 0 \end{aligned}$$

for all permissible *w* and *v*, i.e. all values for which the corresponding values of *t* and *c* are in the support of $f_{T,C}$ for values of $\psi$ and $\lambda$ in their respective parameter spaces. Thus, provided (4.3) is satisfied, any solution to (4.5) is a solution to (4.2) identically in $\psi$, $\lambda$ and $z\ne 0$. If no parameter-free solution to (4.5) exists, there may still be a parameter-dependent solution, as discussed in Sect. 7.1.

In contrast to the original problem (4.2), established solution strategies are available for (4.5), either exact if an analytic solution exists, or approximate. The probabilistic model $f_{T,C}$ may supply additional information to aide solution.

Using the chain rule, the partial differential equation (4.5) can be written

$$\begin{aligned} \frac{\partial s\{t(w,\psi ,\lambda ),c(v,\psi ,\lambda )\}}{\partial t}\frac{\partial t(w,\psi ,\lambda )}{\partial \lambda } {+} \frac{\partial s\{t(w,\psi ,\lambda ),c(v,\psi ,\lambda )\}}{\partial c}\frac{\partial c(v,\psi ,\lambda )}{\partial \lambda } {=} 0. \end{aligned}$$

More compactly, with ${a(t,c):=a(t,c;\lambda ,\psi )=\partial t(w,\psi ,\lambda )/\partial \lambda }$ and $b(t,c):=b(t,c;\lambda ,\psi )=\partial c(v,\psi ,\lambda )/\partial \lambda$,

4.6 $$\begin{aligned} a(t,c)\frac{\partial s(t,c)}{\partial t} + b(t,c)\frac{\partial s(t,c)}{\partial c} = 0. \end{aligned}$$

This is a standard form of partial differential equation to which the method of characteristics applies. See (e.g., [5, Chapter 2]).

The approach extends naturally to $m>2$, although in that case bijectivity of the map implies that *s* has up to $(m-1)$ components and the analogue of (4.1) is

4.7 $$\begin{aligned} f_{S}(s;\psi ,\lambda )=\frac{1}{2\pi i}\int _{\tau - i\infty }^{\tau +i\infty } \cdots \int _{\gamma - i\infty }^{\gamma +i\infty }\exp \{z_1 s_1+\cdots +z_{m-1} s_{m-1}\}T_\lambda (s,z)dz_1\cdots dz_{m-1}, \end{aligned}$$

where, reintroducing subscripts for clarity and assuming the components $Y_{i1},\ldots ,Y_{im}$ are independent, $T_{\lambda }(s,z)$ is

4.8 $$\begin{aligned} \int _{-\infty }^{\infty }\cdots \int _{-\infty }^\infty \exp \{-z_1 s_1-\cdots -z_{m-1} s_{m-1}\} \prod _j f_{Y_{ij}}(y_{ij};\psi _j,\lambda _i)dy_{i1},\ldots , dy_{im}. \end{aligned}$$

The generalisation of (4.3) is that $f_{Y_{ij}}(y_{ij};\psi _j, \lambda _i)$ can be written as

4.9 $$\begin{aligned} f_{Y_{ij}}(y_{ij};\psi _j, \lambda _i)=c_{ij} \frac{d v_{ij}(y_{ij})}{d y_{ij}} g_{ij}\{v_{ij}(y_{ij};\psi _j,\lambda _i),\psi _j\}, \end{aligned}$$

where $c_{ij}$ is a constant and $g_{ij}$ only depends on $\lambda _i$ through the bijective function $v_{ij}(y_{ij};\psi _j,\lambda _i)$.

In principle, these ideas extend beyond matched comparison problems, by replacing $(T_i, C_i)_{i=1}^b$ from the previous discussion by pairs of observations $(Y_i, Y_1)_{i=2}^n$, say, where $Y_{1},\ldots ,Y_n$ are outcome variables whose distribution possibly depends on covariates $x_{1},\ldots ,x_n\in \mathbb {R}^p$. There are connections to maximal invariants used in the theory of invariant tests. Indeed, a referee has pointed out that Example 3 is a location model after a log transformation, so that differences on the log-scale eliminate the nuisance parameter, and that this must be related to the existence of a maximal invariant, or maximal ancillary statistic for ($\lambda _1, \ldots ,\lambda _b$).

Several examples clarify these ideas.

## Matched comparison examples

### Example 4

Suppose that $T_i$ and $C_i$ are independently exponentially distributed of rates $\lambda _i \psi$ and $\lambda _i/\psi$ respectively for $i=1,\ldots ,b$. Example 3 shows that a factorisable transformation exists in the form $S_i=T_i/C_i$. The motivation for the present paper was that this should be recoverable from a seamless application of theory.

Equation (4.2) is, on treating each pair separately and suppressing the subscript on $\lambda$,

5.1 $$\begin{aligned} 0= & {} \frac{\partial }{\partial \lambda }\int _{0}^{\infty }\int _{0}^{\infty } \exp \{-z s(t,c)\} \left\{ \lambda ^{2}\exp (-\lambda \psi t)\exp (-\lambda c/\psi )\right\} dt dc \nonumber \\= & {} \int _{0}^{\infty }\int _{0}^{\infty } \exp \{-z s(t,c)\} \left\{ 2\lambda -\lambda ^2(\psi t + c/\psi )\right\} \exp (-\lambda \psi t)\exp (-\lambda c/\psi ) dt dc.\nonumber \\ \end{aligned}$$

Integration shows that $s(t,c)=t/c$ verifies Eq. (4.2). The goal is to recover this transformation using a strategy that does not require *s*(*t*, *c*) to be known a priori.

Following the recommendation above, change variables in Eq. (5.1) to $w=\lambda \psi t$ and $v=\lambda c/\psi$. The volume element transforms as $dt dc = (\psi \lambda )^{-1} (\lambda /\psi )^{-1}dwdv=\lambda ^{-2}dwdv$ so that Eq. (5.1) is

5.2 $$\begin{aligned} 0 = \frac{\partial }{\partial \lambda }\int _{0}^{\infty }\int _{0}^{\infty } \exp [-z s\{w/(\lambda \psi ),\psi v/\lambda \}] \exp \{-(w+v)\} dw dv. \end{aligned}$$

Dependence on $\lambda$ has been transferred to the bivariate function *s* for which a solution to (5.2) is required. Interchanging the partial differential operator with the integrals shows that a solution to (5.2) is that of the first order linear partial differential equation

5.3 $$\begin{aligned} \frac{\partial }{\partial \lambda } s\{t(w,\psi , \lambda ),c(v,\psi , \lambda )\} =0, \end{aligned}$$

where $t(w,\psi , \lambda )=w/(\lambda \psi )$ and $c(v,\psi , \lambda )=\psi v/\lambda$, and for the purpose of this argument, *w* and *v* should be treated as fixed. Equation (5.3) specifies that ${s:\mathbb {R}^2\rightarrow \mathbb {R}}$ must be constant identically in *v*, *w* and $\psi$ as a function of $\lambda$. Thus, $\{s(t,c)=(t/c)^{k}=(\psi ^{-2}w/v)^k: k\ne 0\}$ is an equivalence class of solutions to (5.3), suggesting $(T_i,C_i) \mapsto \{(T_i/C_i),A\}$ as a suitable transformation, where *A* represents any statistic that makes the transformation bijective, for instance $A=T_i$, $A=C_i$ or $A=T_i C_i$.

### Example 5

Example 4 easily extends to triplets, quadruplets etc. With $X_{i1}$, $X_{i2}$ and $X_{i3}$ exponentially distributed of rates $\lambda _i \psi _1$, $\lambda _i \psi _2$ and $\lambda _i$ respectively, the only change to the previous derivations is that *s* is a function of three variables, which are, on omitting triplet subscripts, $x_1 = w_1/(\lambda \psi _1)$, $x_2 = w_2/(\lambda \psi _2)$ and $x_3=w_3/\lambda$. The equation to be solved is thus (cf Eq. 4.6)

$$\begin{aligned} a(x_1,x_2,x_3)\frac{\partial s(x_1,x_2,x_3)}{\partial x_1} + b(x_1,x_2,x_3)\frac{\partial s(x_1,x_2,x_3)}{\partial x_2} + c(y_1,y_2,y_3)\frac{\partial s(y_1,y_2,y_3)}{\partial x_3} = 0, \end{aligned}$$

say, where $a(x_1,x_2,x_3)=\partial x_1(w_1,\psi _1,\lambda )/\partial \lambda =-x_1/\lambda$, $b(x_1,x_2,x_3)=\partial x_2(w_2,\psi _2,\lambda )/\partial \lambda =-x_2/\lambda$ and $c(x_1,x_2,x_3)=\partial x_3(w_3,\lambda )/\partial \lambda =-x_3/\lambda$. There are multiple solutions. Among those yielding a bijective map from $(x_1,x_2,x_3)$ to $(s_1,s_2,r)$ are $s_1(x_1,x_2,x_3)=x_1/x_3$, $s_2(x_1,x_2,x_3)=x_2/x_3$ and $r=x_3$.

### Example 6

An extension of Example 4 to which the appropriate transformation is not already known has $T_i$ and $C_i$ Weibull distributed of shape $\alpha$ and rate parameters $\lambda _i\psi$ and $\lambda _i/\psi$ respectively. Thus there is one nuisance parameter per pair and another shared over all pairs. The appropriate change of variables is to $w=\lambda \psi t^\alpha$ and $v=(\lambda /\psi )c^\alpha$ so that the analogue of Eq. (5.2) is

$$\begin{aligned} 0 = \frac{\partial }{\partial \lambda }\int _{0}^{\infty }\int _{0}^{\infty } \exp (-z s[\{w/(\lambda \psi )\}^{1/\alpha },\{\psi v/\lambda \}^{1/\alpha }]) \exp \{-(w+v)\} dw dv. \end{aligned}$$

A transformation to eliminate both $\alpha$ and $\lambda$ would need to solve the pair of simultaneous partial differential equations

$$\begin{aligned} a_{\lambda }(t,c)\frac{\partial s(t,c)}{\partial t} + b_{\lambda }(t,c)\frac{\partial s(t,c)}{\partial c}= & {} 0, \\ a_{\alpha }(t,c)\frac{\partial s(t,c)}{\partial t} + b_{\alpha }(t,c)\frac{\partial s(t,c)}{\partial c}= & {} 0, \end{aligned}$$

where ${a_{\lambda }(t,c)= -t/\alpha \lambda }$, ${b_{\lambda }(t,c)= -c/\alpha \lambda }$, ${a_{\alpha }(t,c) = -t\log (t)/\alpha }$, $b_{\alpha }(t,c) = -c\log (c)/\alpha$. There is clearly no such solution. The transformation $s(t,c)=t/c$ eliminates $\lambda$, which is to be favoured over elimination of $\alpha$ because each pair of observations introduces a pair-specific $\lambda _i$. The transformed random variables $(S_{i})_{i=1}^{b}=(T_i/C_i)_{i=1}^{b}$ are independent and identically distributed with probability density function

5.4 $$\begin{aligned} f_S(s;\psi ,\alpha )=\frac{\alpha \psi ^2 s^{\alpha -1}}{(1+\psi ^2s^\alpha )^2}, \quad s>0. \end{aligned}$$

To assess whether it is possible to also eliminate $\alpha$, consider a pair of these transformed random variables, $S_i$ and $S_j$, say. The density function of a transformation $u(s_i,s_j)$ satisfies

$$\begin{aligned} f_U(u;\psi , \alpha )=\frac{1}{2\pi i}\int _{\tau -i\infty }^{\tau + i\infty } \exp \{zu(s_i,s_j)\}T_{\alpha }(u,z)dz, \end{aligned}$$

where

$$\begin{aligned} T_{\alpha }(u,z)=\int _{0}^{\infty }\int _{0}^\infty \exp \{-z u(x,y)\}f_{S_i,S_j}(x,y;\psi ,\alpha ) dx dy, \end{aligned}$$

and analogously to before we seek a solution to $(\partial /\partial \alpha )T_{\alpha }(u,z)=0$. It does not seem possible to specify a change of variables from $(s_i,s_j)$ to (*w*, *v*) such that the dependence on $\alpha$ is transferred from

$$\begin{aligned} f_{S_i,S_j}(s_i,s_j;\psi ,\alpha )= \frac{\alpha \psi ^2 s_i^{\alpha -1}}{(1+\psi ^2s_i^\alpha )^2}\frac{\alpha \psi ^2 s_j^{\alpha -1}}{(1+\psi ^2s_j^\alpha )^2}, \quad s_i>0, s_j>0, \end{aligned}$$

to $u\{s_i(w,\psi ,\alpha ),s_j(v,\psi ,\alpha )\}$. In other words, the analogue of Eq. (4.9) appears to be violated for this relatively complicated form of $f_S(s;\psi ,\alpha )$ given in Eq. (5.4).

The following example, although artificial and having an obvious solution, serves as a reassuring illustration that the distributions involved need not belong to the exponential family.

### Example 7

Suppose that $T_i$ and $C_i$ are independently Cauchy distributed of location $\lambda _i$ and shape $\psi$, the interest parameter. In the notation of Sect. 4,

$$\begin{aligned} T_{\lambda }(s,z)= & {} \int _{-\infty }^\infty \int _{-\infty }^\infty \exp \{-z s(t,c)\}\frac{1}{\pi \psi }\biggl \{1+ \biggl (\frac{t-\lambda }{\psi }\biggr )^2\biggr \}^{-1}\biggl \{1+ \biggl (\frac{c-\lambda }{\psi }\biggr )^2\biggr \}^{-1} dt dc \\= & {} \frac{1}{\pi ^2} \int _{-\infty }^\infty \int _{-\infty }^\infty \exp \{-zs(\psi w+\lambda , \psi v + \lambda )\}\frac{dw dv}{(1+w^2)(1+v^2)}, \end{aligned}$$

where we have used a change of variables to $w=(t-\lambda )/\psi$ and $v=(c-\lambda )/\psi$. The function $s:\mathbb {R}^2\rightarrow \mathbb {R}$ must be constant identically in *v*, *w* and $\psi$ as a function of $\lambda$ showing that any $\lambda$-free function of $(t-c)=\psi (w-v)$ is a solution to (4.5).

## Further examples

The strategy extends beyond the block structure of the previous examples, or equivalently to settings with *b* blocks of size one or a single block of size *n*.

### Example 8

. Let $Y_{1},\ldots ,Y_{n}$ be exponentially distributed with means $\mathbb {E}(Y_{i})=\lambda \exp (x_i^{\mathrm{T}}\psi )$ for covariates $x_i\in \mathbb {R}^p$. A simplified version of this example was studied by [10] from a different perspective. For an arbitrary pair $(Y_{i},Y_{1})$, say:

$$\begin{aligned} T_{\lambda }(s,z) = \int _{-0}^\infty \int _{-0}^\infty \exp \{-z s(\lambda \exp (x_i^{\mathrm{T}}\psi )w_i,\lambda \exp (x_1^{\mathrm{T}}\psi )w_1)\}\exp \{-(w+v)\} dw_i dw_1, \end{aligned}$$

where we have used a change of variable from $y_i$ to $w_i=\lambda ^{-1}\exp (-x_i^{\mathrm{T}}\psi )y_i$.

The differential equation to be solved in $s(y_{j},y_{1})$ is

$$\begin{aligned} 0= & {} \frac{\partial s\{y_{i}(w_i,\lambda ,\psi ), y_{1}(w_1,\lambda ,\psi )\}}{\partial \lambda } \\= & {} w_i(y_i, \psi ,\lambda ) \exp (x_i^{\mathrm{T}}\psi ) \frac{\partial s(y_{i},y_{1})}{\partial y_{i}} + w_1(y_1, \psi ,\lambda )\exp (x_1^{\mathrm{T}}\psi ) \frac{\partial s(y_{i},y_{1})}{\partial y_{1}}, \end{aligned}$$

for which solutions are of the form $\{s(y_{i},y_{1})=(y_{i}/y_{1})^k:k\ne 0\}$. Thus, inference based on $(Y_{i}/Y_{1})_{i=1}^n$ is free of the nuisance parameter $\lambda$.

### Example 9

([8], Sect. 11) discussed accelerated life models as convincing alternatives to proportional hazards representations in some contexts. One convenient parametric form, particularly when censoring is present, is the log-logistic model (e.g. [14, p. 190]), which has a potentially non-monotonic hazard function. Let $Y_{i}$ be a log-logistic random variable with shape parameter $\beta >0$, determining monotonicity or otherwise, and scale parameter $\alpha _i=\lambda \exp (x_i^{\mathrm{T}}\psi )$ for $\lambda >0$. The density function of $Y_{i}$ is given by

$$\begin{aligned} \frac{(\beta /\alpha _i)(y/\alpha _i)^{\beta -1}}{(1+(y/\alpha _i)^\beta )^2}, \quad y>0. \end{aligned}$$

Solutions to the differential equation associated with an arbitrary pair $(Y_{i},Y_{1})$ are of the form $\{s(y_{i},y_{1})=(y_{i}/y_{1})^k:k\ne 0\}$, again showing that inference based on $(Y_{i}/Y_{1})_{i=1}^n$ is free of the nuisance parameter $\lambda$.

## Extending applicability

The previous analysis was limited by two aspects: that the marginal likelihood factorisation is not universally valid and indeed may only hold in rather special cases, such as models built on transformation groups; secondly, the requirement (4.3), which arises from the solution strategy for (4.2). It seems likely that applicability of the ideas of Sect. 4 can be extended, and we outline some possible routes to this.

### Perturbed factorisations

Existence of a marginal likelihood factorisation can be relaxed by supposing that such a factorisation is approximately valid, with the factor purported to depend only on $\psi$ in fact depending weakly on $\lambda$. If this more general condition is satisfied, $\lambda$ can be replaced by an arbitrary value without materially affecting inference for $\psi$.

The relaxation can be incorporated by specifying that the right hand side of (4.5) is not exactly zero but rather a slowly varying function, *h* say, of $\lambda$. In the context of Sect. 4, the resulting equation is

7.1 $$\begin{aligned} \frac{\partial }{\partial \lambda } s\{t(w,\psi , \lambda ),c(v,\psi , \lambda )\} = h(\lambda ), \end{aligned}$$

and since $h(\lambda )$ is unknown, one approach is to expand it locally around a base point, $\lambda _0$, leading to the approximation

$$\begin{aligned} \frac{\partial }{\partial \lambda } s\{t(w,\psi , \lambda ),c(v,\psi , \lambda )\}= & {} a(t,c)\frac{\partial s(t,c)}{\partial t} + b(t,c)\frac{\partial s(t,c)}{\partial c} \\= & {} \kappa + \varepsilon _1 (\lambda - \lambda _0)+\varepsilon _2 (\lambda - \lambda _0)^2, \end{aligned}$$

where $\kappa$ is a constant, $\varepsilon _1$ and $\varepsilon _2$ are small and, as explained in Sect. 4, $a(t,c)=\partial t(w;\psi ,\lambda )/\partial \lambda$ and $b(t,c)=\partial c(v;\psi ,\lambda )/\partial \lambda$. This is now a first-order linear inhomogenous differential equation that can, in principle, be solved by the method of characteristics when $\kappa$, $\lambda _0$, $\varepsilon _1$ and $\varepsilon _2$ are treated as known. Provided that the limit of the resulting solution as $\varepsilon _1,\varepsilon _2 \rightarrow 0$ is operational for small $(\lambda -\lambda _0)$, this specifies a transformation that is approximately factorisable in the earlier sense.

An alternative, simpler, route is to seek a solution to the original homogeneous equation $(\partial /\partial \lambda ) s\{t(w,\psi , \lambda ),c(v,\psi , \lambda )\}=0$, acknowledging that the transformation *s*(*t*, *c*) will in general depend on the unknown parameters. If such a solution depends on $\psi$ only, the distribution of $s(T,C;\psi )$ is free of $\lambda$ at the true value of $\psi$. This leads to a procedure for constructing confidence sets for $\psi$ analogous that proposed by [3] in the context of conditional likelihood. Specifically, any $\psi _0$ for which the sample of suitably standardised statistics $s(t,c;\psi _0)$ is consistent with its theoretical distribution, assuming $\psi _0=\psi$, constitutes a confidence set for $\psi$.

In reducing or eliminating dependence on the nuisance parameter, it is possible that dependence on the interest parameter is also weakened to the extent that marginal likelihood is ineffective. This is a limitation of the procedure which needs to be checked for.

#### Example 10

Let $T_i$ and $C_i$ for $i=1,\ldots ,b$ be independently exponentially distributed of means $\mu _i - \psi$ and $\mu _i+\psi$ respectively, or equivalently of rates $\lambda _i/(1-\lambda _i\psi )$ and $\lambda _i/(1+\lambda _i\psi )$, where $-1<\lambda _i\psi <1$. There is no obvious choice of partial likelihood function.

Dropping subscripts, the appropriate change of variables is to $w=t\lambda /(1-\lambda \psi )$ and $v=c\lambda /(1+\lambda \psi )$, therefore

$$\begin{aligned} a(t,c):= & {} \frac{\partial t(w;\psi ,\lambda )}{\partial \lambda } = -\frac{w}{\lambda ^2} = \frac{t}{\lambda (1-\lambda \psi )}, \\ b(t,c):= & {} \frac{\partial c(v;\psi ,\lambda )}{\partial \lambda } = -\frac{v}{\lambda ^2} = \frac{c}{\lambda (1+\lambda \psi )}. \end{aligned}$$

A set of solutions to the resulting homogeneous equation,

$$\begin{aligned} \frac{t}{\lambda (1-\lambda \psi )} \frac{\partial s(t,c)}{\partial t} + \frac{c}{\lambda (1+\lambda \psi )}\frac{\partial s(t,c)}{\partial c} = 0, \end{aligned}$$

is $\{s(t,c)=(c/t^{\beta })^k : k\ne 0\}$, where $\beta =(1-\lambda \psi )/(1+\lambda \psi )$, and it is convenient to take $k=1$. The transformation depends on the unknown parameters $\lambda$ and $\psi$, and *T*/*C* provides a crude estimate of $\beta$ so that $C/T^{T/C}$ is one candidate transformation.

Instead consider a leading order Taylor series expansion around $\psi \lambda =0$. This gives $s(t,c)\approx \tilde{s}(t,c)=c/t$. Thus $\tilde{s}(t,c)=c/t$ is the parameter-free transformation whose marginal density function depends only weakly on $\lambda$ provided that $\psi \lambda$ is small. The latter condition is not unreasonable because the formulation requires $-1< \psi \lambda < 1$.

However, direct calculation shows that the density function of $\tilde{S}=C/T$ is determined by the product $\psi \lambda$ as

7.2 $$\begin{aligned} f_{\tilde{S}}(s;\psi ,\lambda ) = \frac{1-(\psi \lambda )^2}{\{1+s + \psi \lambda (1-s)\}^2}, \quad s>0. \end{aligned}$$

**Fig. 1:**
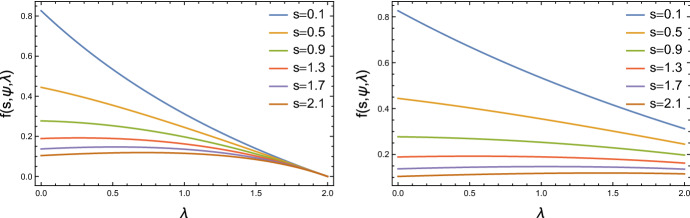
Plot of Eq. (7.2) as a function of $\lambda$ for $\psi =0.5$ (left), $\psi =0.25$ (right), and for the specified values of *s*

Thus, although the transformation has been effective in approximately eliminating dependence on $\lambda$ for any given $\psi$, as illustrated in Fig. 1, a partial likelihood function based on (7.2) caries information only about the product $\psi \lambda$. We have found instead an approximately ancillary statistic for $(\psi ,\lambda )$. Further discussion of this example is in Sect. 8.

### Other solution strategies for the integro-differential equation (4.2)

A further limitation of the proposal in Sect. 4 is the requirement (4.3). This arises from the solution strategy for (4.2) in terms of a partial differential equation. In developing the ideas from Sect. 4, we considered several other solution strategies. While we were unable to fully operationalise them, it seems valuable to report them here as possible routes for further exploration. We illustrate these ideas in the context of Example 4.

#### Change of variables after differentiation under the integral sign

A re-expression of the second equation in (5.1), in which the differentiation has been performed under the integral sign, is

7.3 $$\begin{aligned} 0 = \int _{0}^{\infty } \exp (-\lambda c/\psi ) \left[ \int _{0}^{\infty }\exp \{-z s(t,c)\}\{2\lambda - \lambda ^2(\psi t+c/\psi )\}\exp (-\lambda \psi t) dt \right] dc. \end{aligned}$$

Thus any *s*(*t*, *c*) that makes the function in square brackets orthogonal to $\exp (-\lambda c/\psi )$ identically in $\lambda$, $\psi$ and *z*, also solves (5.1).

By changing variables to $x=\sqrt{c}$, (7.3) is

$$\begin{aligned} 0 = \int _{-\infty }^{\infty } \frac{\exp (-\lambda x^2/\psi )}{2x} \left[ \int _{0}^{\infty }\exp \{-z s(t,x^2)\}\{2\lambda - \lambda ^2(\psi t+x^2/\psi )\}\exp (-\lambda \psi t) dt \right] dx. \end{aligned}$$

and any $s(t,x^2)$ that makes the term in square brackets an even function, identically in *z*, $\psi$ and $\lambda$ satisfies the equation, because $\exp (-\lambda x^2/\psi )$ is an even function and 1/2*x* is odd.

#### Hilbert–Schmidt orthogonalisation after differentiation under the integral sign

From any linearly independent functions $f_0,\ldots ,f_K$, orthogonal functions $\phi _0,\ldots ,\phi _K$ can be constructed as described by [29, Chapter II] or Whittaker andWatson ([31], Sect. 11.6). Thus, in view of (7.3), set

$$\begin{aligned} \phi _0(c) = f_{0}(c) = \exp (-\lambda c/\psi ). \end{aligned}$$

For any $\kappa \ne 1$, $f_{1}(c)=\exp (-\lambda \kappa c/\psi ):= f_{1}^{(\kappa )}(c)$ is linearly independent of $f_0$, and a family of functions $\phi ^{(\kappa )}_{1}$, orthogonal to $\phi _{0}$ can be constructed as [29, Chapter II]

7.4 $$\begin{aligned} \phi _{1}^{(\kappa )}(c)=(D_0 D_1^{(\kappa )})^{-1/2}D_{1}^{(\kappa )}(c), \end{aligned}$$

where $$D_0 := \langle f_0,f_0 \rangle $$, and

$$\begin{aligned} D_{1}^{(\kappa )}(c):= & {} \langle f_0,f_0 \rangle f_1^{(\kappa )}(c) - \langle f_0,f_1^{(\kappa )}\rangle f_0(c), \\ D_1^{(\kappa )}:= & {} \langle f_0,f_0\rangle \langle f_1^{(\kappa )},f_1^{(\kappa )}\rangle - \langle f_0,f_1^{(\kappa )}\rangle ^{2}, \end{aligned}$$

and for any pair of functions *f* and *g*, each in $\mathbb {L}_{2}(a,b)$, $\langle f,g \rangle =\int _{a}^{b} f(x)g(x) dx$.

For any $\kappa >0$, with the $f_0$ and $f_1^{(\kappa )}$ defined above,

$$\begin{aligned} \langle f_0,f_1^{(\kappa )}\rangle= & {} \int _{0}^{\infty }\exp (-\lambda c/\psi )\exp (-\lambda \kappa c/\psi ) dc = \psi /(\lambda + \kappa \lambda ), \\ \langle f_1^{(\kappa )},f_1^{(\kappa )} \rangle= & {} \int _{0}^{\infty }\exp (-\lambda \kappa c/\psi )\exp (-\lambda \kappa c/\psi ) dc = \psi /(2 \kappa \lambda ), \end{aligned}$$

and $$(f_0,f_0)=\psi /(2\lambda )$$. Thus

$$\begin{aligned} (D_0 D_1^{(\kappa )})^{-1/2} = \frac{2\sqrt{2}\kappa ^{1/2}(\kappa + 1)\lambda ^{3/2}}{(\kappa -1)\psi ^{3/2}}, \end{aligned}$$

and using Eq. (7.4),

$$\begin{aligned} \phi _{1}^{(\kappa )}(c) = \frac{\sqrt{2\kappa \lambda }(\kappa +1)}{(\kappa -1)\psi ^{1/2}}\exp (-\lambda \kappa c/\psi ) - \frac{2\sqrt{2\kappa \lambda }}{(\kappa -1)\psi ^{1/2}}\exp (-\lambda c/\psi ). \end{aligned}$$

It can be checked by integration that $\int _{0}^{\infty }\phi _{0}(c) \phi _{1}^{(\kappa )}(c)dc = 0$. It follows that a family of solutions to (7.3) are the functions *s*(*t*, *c*) that solve

$$\begin{aligned} \phi _{1}^{(\kappa )}(c) = \int _{0}^{\infty }\exp \{-z s(t,c)\}\{2\lambda - \lambda ^2(\psi t+c/\psi )\}\exp (-\lambda \psi t) dt. \end{aligned}$$

#### Stein operators

Consider operators $\mathcal {A}_{Q}$, which characterize a distribution *Q* in the sense that

7.5 $$\begin{aligned} \mathbb {E}_{Q}(\mathcal {A}_{Q}f)(X) = 0 \quad \forall f \in \mathcal {F} \;\; \Longleftrightarrow \;\; X \sim Q, \end{aligned}$$

where $\mathcal {F}$ is the space of smooth and bounded functions. Stein [28] showed that for *Q* the standard normal distribution, the operator $A_{Q}$ is $(\mathcal {A}_{Q}f)(x)=f^{\prime }(x) - xf(x)$. For *Q* an exponential distribution of rate $\rho$, the corresponding $\mathcal {A}_{Q}$ is [26],

7.6 $$\begin{aligned} (\mathcal {A}_{Q}f)(x)=(1-\rho x)f^{\prime }(x) + xf^{\prime \prime }(x). \end{aligned}$$

Let $w(c;\psi ,\lambda )$ denote the function in square brackets in (7.3). Since (7.5) is a complete characterisation of *Q*, any function *w* satisfying (7.3) also satisfies

$$\begin{aligned} (\psi /\lambda )w(c;\psi ,\lambda ) = (1-\lambda c/\psi )f^\prime (c) + cf^{\prime \prime }(c) \end{aligned}$$

for some smooth and bounded function *f*, and so the equation to be solved for *s*(*t*, *c*), identically in *z*, $\lambda$ and $\psi$, is

$$\begin{aligned}&(1-\lambda c/\psi )f^\prime (c;z,\lambda , \psi ) + cf^{\prime \prime }(c;z,\lambda , \psi )\\&\quad = \lambda \psi ^{-1}\int _{0}^{\infty }\exp \{-z s(t,c)\}\{2\lambda - \lambda ^2(\psi t+c/\psi )\}\exp (-\lambda \psi t) dt \end{aligned}$$

for any convenient choice of $f\in \mathcal {F}$.

## Reducing the role of nuisance parameters through other routes

While complete elimination of nuisance parameters is typically highly effective when available, other routes to reducing their role may sometimes be more fruitful.

Rather than seeking a transformation whose distribution is free of the nuisance parameters, we may instead seek one whose expectation is free of them, a more modest goal, leading to empirical averages as point estimators. In principle this should be easier to operationalise in a systematic way than complete elimination from the distribution of the transformation, although we have not obtained a unifying formulation. The variance of this point estimator in general depends on the nuisance parameters, and the challenge is then to find an accurate estimate of the composite, thereby evading separate estimation of each one.

### Example 11

Suppose that $T_i$ and $C_i$ are as in Example 10. Then

$$\begin{aligned} \hat{\psi }:=\frac{1}{2b}\sum _{i=1}^b (C_i-T_i) \rightarrow _p \frac{1}{2b}\sum _{i=1}^b \mathbb {E}(C_i-T_i) = \psi \end{aligned}$$

is a consistent point estimator as $n\rightarrow \infty$. The variance of $\hat{\psi }$, unsurprisingly, depends on all *b* nuisance parameters. In particular

$$\begin{aligned} \text {var}(\hat{\psi })=\frac{\psi ^2}{2b} + \frac{1}{2b^2}\sum _{i=1}^b \frac{1}{\lambda _i^2}. \end{aligned}$$

However, since

$$\begin{aligned} \frac{1}{b}\sum _{i=1}^b T_iC_i \rightarrow _p \frac{1}{b}\sum _{i=1}^b \mathbb {E}(T_i C_i) = \frac{1}{b}\sum _{i=1}^b \frac{1}{\lambda _i^2} - \psi ^2, \end{aligned}$$

a consistent estimator of the variance of $\hat{\psi }$ is

$$\begin{aligned} \hat{\sigma }^2:= \frac{1}{2b^2}\sum _{i=1}^b T_i C_i + \frac{\hat{\psi }^2}{b} \rightarrow _p \frac{\psi ^2}{2b} + \frac{1}{2b^2}\sum _{i=1}^b \frac{1}{\lambda _i^2}. \end{aligned}$$

Although there are compelling reasons for preferring likelihood-ratio inference in low dimensions, Example 10 is a case for which direct use of the likelihood is not recommended, and for which partial likelihood may be infeasible, while a pivotal quantity $(\hat{\psi }-\psi )/\hat{\sigma }$ can be derived from simple algebraic operations. Following [30], confidence sets can in principle be constructed from this quantity, although its approximate normality requires investigation, as the usual regularity conditions do not hold.

In Example 11, the original nuisance parameters are neither estimated nor eliminated. Instead, an implicit reparametrisation is performed, producing a scalar composite nuisance parameter. Accumulation of estimation error from multitudinous nuisance parameters is thereby avoided. This points to a more general strategy, in principle applying even when $p>n$, in which transformations are sought to make the problem depend on the interest parameter and, at most, a small set of one-dimensional summaries of the original nuisance parameters.

## Closing remarks

In an earlier related paper [4], we closed with some open problems having a differential geometrical bearing. The first of these questioned whether a connection could be established between data-based transformations for the elimination of nuisance parameters via marginal or conditional likelihood, and the interest-respecting reparameterisations of [10]. The following remarks are informal.

Fraser [17] made a connection between the sample and parameter spaces through the notion of a local location model. The parameterised distribution function $F_{Y}(y;\theta )$ of *Y*, say, is viewed a function of both *y* and the parameter $\theta$. Let $\varepsilon$ be a quantile defined by $F(y_{\varepsilon }(\theta );\theta )=\varepsilon$. Then

$$\begin{aligned} 0 = \frac{\partial F (y_{\varepsilon }(\theta );\theta )}{\partial y_{\varepsilon }}\frac{\partial y_{\varepsilon }(\theta )}{\partial \theta } + \frac{\partial F(y_{\varepsilon };\theta )}{\partial \theta }, \end{aligned}$$

and since $\varepsilon$ is arbitrary

$$\begin{aligned} \frac{\partial y (\theta )}{\partial \theta } = - \frac{\partial F(y;\theta )/\partial \theta }{f(y;\theta )}. \end{aligned}$$

The exposition in [17] is different.

These ideas have been fruitfully employed in the development of the tangent exponential model, starting with [19, 20]. Davison and Reid [15] provide a summary aries from a different perspective with more detailed accounts of the historical development.

We close with an acknowledgement of the limitations of this work. The strategy we sought to follow was to start from some examples for which we already knew the answer and to find a general theory that recovers those answers. Where the paper falls short is in its extension of this theory to other structures, raising two possibilities: either that we have not taken full advantage of our ideas, or that they are less general than we would hope. Which of these possibilities is true can only be ascertained after further attempts at development, possibly along the lines of Sect. 7.

## Data Availability

There are no data associated with this work.

## Treating nuisance parameters as fixed or random

The next two paragraphs follow an exposition due to [24]. Let ($\mathcal {Y},\mathcal {A}$) denote the common Borel space for the independent random variables $Y_1,\ldots ,Y_b$. For each *i*, $Y_i$ has a parametric density function $f(y; \psi , \lambda _i)$ with respect to $\sigma$-finite measure on ($\mathcal {Y},\mathcal {A}$), known up to $\psi$ and $\lambda _i$, where $(\psi ,\lambda _i)$ is assumed to belong to the Cartesian product parameter space $\Theta =\Psi \times \Lambda$ so that $\psi$ and $\lambda _i$ are variation independent. Let $\prod _{i=1}^b (\psi ,\lambda _i)$ denote the measure on ($\mathcal {Y}^b,\mathcal {A}^b$) induced by $(Y_1,\ldots ,Y_b)$.

A key consideration is whether the nuisance parameters $\lambda _1,\ldots ,\lambda _b$ are treated as realisations of random variables or as fixed arbitrary constants. Lindsay [24] formalised the distinction by introducing the mixture model

$$\begin{aligned} f(y;\psi , Q)= \int f(y;\psi ,\lambda )dQ(\lambda ), \end{aligned}$$

say, whose induced measure on $(\mathcal {Y}^b,\mathcal {A}^b)$ is denoted by $(\psi , Q)^b$. Let $\delta (\lambda )$ be the probability measure assigning mass 1 to $\{\lambda \}$. Lindsay [24] noted that the parameter space $\Theta$ can be embedded in

$$\begin{aligned} \Theta ^* := \{(\psi ,Q): \psi \in \Psi , Q\in \mathcal {Q}\} \end{aligned}$$

provided that the space of probability measures $\mathcal {Q}$ is sufficiently rich to include each of $\delta (\lambda _1), \ldots ,\delta (\lambda _b)$. In practice, $\mathcal {Q}$ is typically taken as a parametric family, although Kiefer and Wolfowitz (1956) allowed an infinite-dimensional $\mathcal {Q}$ under some regularity conditions.

This raises important conceptual issues that go to the foundations of modelling. While, in principle, $\Theta \subseteq \Theta ^*$ when $\mathcal {Q}$ is unrestricted, to treat $\lambda _1, \ldots ,\lambda _b$ as being drawn from a non-degenerate distribution rather than as fixed unknown constants is an extra assumption whose implications may be minor or considerable depending on context. A more extreme version of essentially the same problem is the weighing machine example of [6].

The random effects formulation, if it is to be believed, has the appealing feature that $\psi$ and *Q* together capture stable aspects of the system and are therefore relevant for predicting future observations. For scientific understanding via $\psi$, lack of stability of the model with respect to the nuisance parameters $\lambda _1,\ldots ,\lambda _b$ is immaterial except insofar as it might affect inference on $\psi$. For the matched comparison studies that motivate the present paper, whereby $\psi$ represents a treatment effect and $\lambda _1,\ldots ,\lambda _b$ capture block-specific effects, the conceptual motivation for treating these as fixed unknown constants seems strong and leads, at least in principle, to an answer free of assumptions about the inter-block variation.
